# Genomic New Insights Into Emergence and Clinical Therapy of Multidrug-Resistant *Klebsiella pneumoniae* in Infected Pancreatic Necrosis

**DOI:** 10.3389/fmicb.2021.669230

**Published:** 2021-06-25

**Authors:** Haibin Hao, Yang Liu, Jin Cao, Kun Gao, Yingying Lu, Weiping Wang, Peng Wang, Sida Lu, Long Hu, Zhihui Tong, Weiqin Li

**Affiliations:** ^1^Department of Critical Care Medicine, Affiliated Jinling Hospital, Medical School of Nanjing University, Nanjing, China; ^2^Laboratory of Microbiology, Basic Medical Laboratory, Affiliated Jinling Hospital, Medical School of Nanjing University, Nanjing, China; ^3^School of Medicine, Nanjing Medical University, Nanjing, China; ^4^School of Medicine, Southeast University, Nanjing, China; ^5^Department of Bioinformatics, Hugobiotech Co., Ltd., Beijing, China; ^6^National Institut of Healthcare Data Science at Nanjing University, Nanjing, China; ^7^State Key Laboratory of Pharmaceutical Biotechnology, School of Life Sciences, Nanjing University, Nanjing, China

**Keywords:** whole genome sequencing, multi-drug resistant, *Klebsiella pneumoniae*, ceftazidime-avibactam, aztreonam, infected pancreatic necrosis

## Abstract

Infected pancreatic necrosis (IPN) is a key risk factor in the progression of severe acute pancreatitis, and use of antibiotics is one of the main clinical actions. However, early prophylactic or unreasonable use of antibiotics promotes drug resistance in bacteria and also delays optimum treatment. To explore genomic evidence of rational antibiotic use in intensive care units, we isolated *Klebsiella pneumoniae* from IPN samples that showed the highest positive-culture rate in 758 patients. Based on whole-genome sequencing from eight strains, 42 antibiotic-resistant genes were identified in the chromatin and 27 in the plasmid, which included classic resistance-mechanism factors such as β-lactamases [16.67% (7/42) in the chromatin and 25.93% (7/27) in the plasmid]. The *K. pneumoniae* isolates were identified to be resistant to multiple antibiotics used in clinics. *In vivo* and *in vitro*, ceftazidime-avibactam (CZA) plus aztreonam (ATM) (2.5:1) showed more significant antibacterial effectiveness than CZA alone. The isolated *K. pneumoniae* were of three different types according to the resistance phenotypes for CZA and ATM. Those co-harboring *bla*_NDM–5_, *bla*_CTX–M–15_, *bla*_OXA–1_, and *bla*_SHV–187_ showed higher resistance to CAZ than *bla*_NDM–5_. Those co-harboring *bla*_CTX–M–65_, *bla*_SHV–182_, and *bla*_TEM–181_ were significantly less resistant to β-lactam than to other extended-spectrum β-lactamases. However, β-lactamases were inhibited by avibactam (AVI), except for NDM-5. ATM plus AVI showed a significant inhibitory effect on *K. pneumoniae*, and the minimum dosage of ATM was < 1 mg/L. In conclusion, we propose that ATM plus AVI could be a major therapy for complex infectious diseases caused by multidrug-resistant *K. pneumoniae*.

## Graphical Abstract

Based on the genotype and phenotype, aztreonam plus avibactam were considered for multi-drug resistant *Klebsiella pneumoniae* in infected pancreatic necrosis.

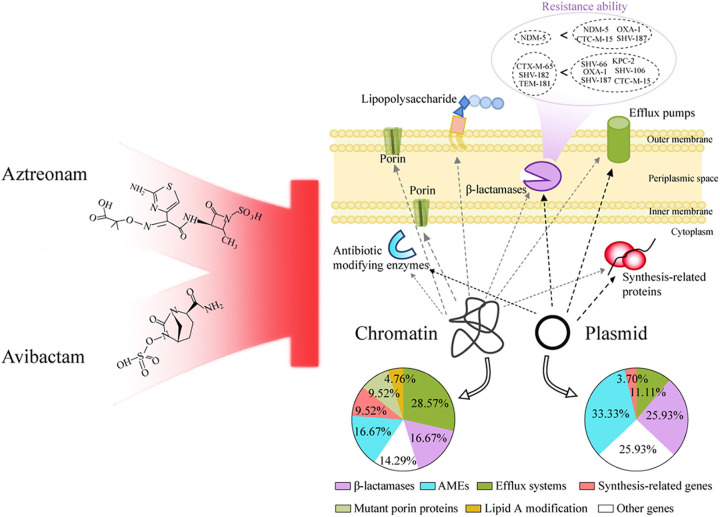

## Introduction

The prevalence of multidrug-resistant (MDR) *Klebsiella pneumoniae* is increasing worldwide (van Duin et al., 2020), and infection control has not been ideal in intensive care units (ICUs). Further, during the ongoing coronavirus disease 2019 (COVID-19) pandemic, the treatment of MDR *K. pneumoniae* has become more complicated. Almost 70% of COVID-19 patients in ICUs were treated with broad-spectrum antibiotics, and more than 70% of positive-culture microbial strains, including MDR *K. pneumoniae*, were resistant to these antibiotics (Arteaga-Livias et al., 2021; Gomez-Simmonds et al., 2021). Moreover, COVID-19 patients infected by MDR *K. pneumoniae* had high risk of bacteremia (Hosoda et al., 2021), and some COVID-19-related death cases were confirmed to have multiple infections by MDR *K. pneumoniae* (Arcari et al., 2021). Therefore, patients in ICUs, especially COVID-19 patients, must be administered timely, reasonable, and accurate anti-infection treatment for MDR *K. pneumoniae*.

Ceftazidime-avibactam (CZA) is a new cephalosporin β-lactamase inhibitor with an activity spectrum against gram-negative bacteria, approved by the United States in 2015, the European Union in 2016, and China in 2019. Increasingly more bacteria have been clinically found to be resistant to CZA (Zhang et al., 2020). Using CZA plus other antibiotics, such as CZA plus metronidazole or meropenem or aztreonam (ATM), to achieve antibacterial effects has become an alternative strategy. Similarly, CZA plus ATM has been used occasionally in our hospital to treat pancreatic infections and has been found to be effective. However, this strategy lacks genomic evidence and could have the potential to enhance the resistance determinants. Indeed, metallo-β-lactamase-producing *K. pneumoniae* have shown resistance to CZA plus meropenem/vaborbactam (Dulyayangkul et al., 2020). Recently, ATM plus avibactam (AVI) has been shown to be an effective alternative to CZA for inhibiting MDR bacteria, but it has not yet been applied in the clinical setting (Wei et al., 2020).

The treatment center for severe acute pancreatitis in the ICU of our hospital has emerged as an important pancreatitis referral center in China (Gao et al., 2018). We receive more than 200 patients with severe acute pancreatitis each year from ICUs across the country. During the ongoing COVID-19 pandemic, our team often faces great trials and pressures, since infections are one of the leading causes of severe acute pancreatitis-related death (Ding et al., 2019). In this study, to avoid overuse and misuse of antibiotics, we first analyzed bacteria distribution and drug-resistance characteristics and then attempted to elucidate the underlying molecular mechanism using whole-genome sequencing (WGS). We reveal new genomic insights and show that ATM plus AVI may be the most suitable strategy for treating complex infectious diseases caused by MDR *K. pneumoniae* in patients in ICUs.

## Materials and Methods

### Clinical Data and Strains

We reviewed all the patients with AP who developed IPN between January 2015 and December 2019 in the Treatment Center of Severe Acute Pancreatitis of Jinling Hospital and analyzed the distribution of pathogens from pancreatic or peripancreatic necrosis. The necrotic samples were collected by fine-needle aspiration, percutaneous drainage, or open surgery. Patients with at least one positive culture were included. If several positive cultures were obtained from one patient during the same admission, only different bacteria were included. All the data were extracted from a prospectively collected database (AP Database, partial technical support from MedicalSystem Company of Suzhou in China) approved by the Ethics Committee of the Affiliated Jinling Hospital, Medical School of Nanjing University. Written informed consent was obtained from all patients or their next of kin during admission for medical data collection, storage, and scientific application after desensitization.

Eight strains of K. pneumoniae were isolated from eight patients with IPN to perform WGS between August 2020 and September 2020. These patients were admitted to our ICU after the COVID-19 pandemic was under control in China. Most importantly, the anti-infective efficacy of CZA was not ideal for them, which was the main reason to perform WGS.

### Antimicrobial Susceptibility Testing

Antimicrobial susceptibility testing (AST) of 758 patients with IPN were performed by the Department of Microbiology between 2015 and 2019, and we retrospectively analyzed these data. Eight strains of *K. pneumoniae* were isolated from eight patients with IPN between August 2020 and September 2020. In the Department of Microbiology, Mueller-Hinton agar plates were used as the culture medium for the isolation of *K. pneumoniae*, and WGS was performed from a single colony of *K. pneumoniae* on a Mueller-Hinton agar after 24 h of incubation. Based on the results of the antimicrobial susceptibility testing (AST), which was performed precisely according to the protocols of the Clinical and Laboratory Standards Institute (CLSI) M100 (CLSI, 2020), they were classified as the multi-drug resistance bacteria by the Microbiology Department of Jinling Hospital with reference to CLSI resistant breakpoints. Antibiotics AST kits were used, such as Ceftazidime (catalog number: J13004, Kangtai Biological Technology Co., Ltd., Wenzhou, China), Cefepime (J13008, Kangtai), Cefazolin (J13006, Kangtai), Ceftriaxone (J13002, Kangtai), Cefotetan (CT0665B, Thermo Scientific^TM^ Oxoid^TM^, United Kingdom), Cefuroxime Axetil (231620, BD Biosciences, United States), Cefuroxime Sodium (CT0127B, Thermo Scientific^TM^ Oxoid^TM^, United Kingdom), Ceftazidime-Avibactam (S21121, Kangtai), Cefoperazone-Sulbactam (Z51042, Kangtai), Piperacillin (J13009, Kangtai), Piperacillin-Tazobactam (J13044, Kangtai), Imipenem (J13049, Kangtai), Meropenem (J13050, Kangtai), Aztreonam (J13048, Kangtai), Levofloxacin (J13016, Kangtai), Ciprofloxacin (J13015, Kangtai), Compound Sulfamethoxazole (J13045, Kangtai), Gentamicin (J13021, Kangtai), Tobramycin (J13051, Kangtai), Amikacin (J13046, Kangtai), Ampicillin-Sulbactam (J13043, Kangtai), Tigecycline (Z51079, Kangtai), and Polymyxin B (J1A086, Kangtai).

### WGS

The illumina data from next-generation sequencing was filtered from low-quality data using FastP v0.20.1 to get clean reads (Bush, 2020), and the reference genome (GCF_001887985.3_ASM188798v3) was indexed using the bwa index command of BWA v0.7.17^1^. Samtools-1.11 was used to remove the duplicates in one BAM file. The HaplotypeCaller module of Genome Analysis Toolkit (GATK) v4.1.9.0 was used to detect SNPs/indels, and the VariantFiltration module was used to filter SNPs/indels and calculate differences in numbers among the samples and between the samples and the reference. The number of SNPs/indels was used to construct a phylogenetic tree using UPGMA methods based on MEGA v10.2.0.

The nanopore data from third-generation sequencing was filtered from low quality data using Filtlong v0.2.0^2^, and the barcode was cut out with internal software. The minimum length was 500 bp and the lowest average quality of the reads was 8. Seqtk v1.3 was used to extract approximately 100 × of illumina data and 15 × of nanopore data, which were mixed and assembled in the genome using Unicycler v0.4.8, and the min_fasta_length was 500 bp. The drug resistance and virulence databases in the assembled genome sequence were annotated using ABRicate v1.0.1^3^, with a minimum consistency of 90 and a minimum coverage of 60. Genome annotation was performed using Prokka v1.14.5.

### Bacterial Growth Curve Analysis

After forming colonies on LB agar plates, at least three colonies were incubated in 25 mL LB at 37°C with 200 rpm shaking. During the incubation, 200 μL of the bacterial solution was transferred to a 96-well plate every 2 h up to 24 h, and two repeats were performed for every colony. Bacterial growth was measured as absorbance at 600 nm (Abs600nm) using a Multiskan GO plate reader (Thermo Fisher Scientific, United States), and then the 24 h growth curve of *K. pneumoniae* was obtained. The bacterial solution of *K. pneumoniae* incubated for 24 h was measured at Abs600nm after gradient dilution to finally get the standard curve of various concentration atAbs600nm, ensuring the right experimental conditions. *Escherichia coli* DH5α was used as the control.

### Drug Inhibition Assay

On studying the 24-h growth curve of *K. pneumoniae*, the 6-h incubation time was observed to display a relatively good logarithmic growth phase at Abs600nm value of 0.3. Based on the CLSI guidelines, the bacterial solution was diluted to 0.5 McFarland turbidity, and the sensitivity to antibiotics was assessed using the broth microdilution method. After 1:100 dilution, 100 μL of the bacterial solution in LB medium was added to each well in a 96-well plate, along with 100 μL of gradient-diluted antibiotics, to reach a total volume of 200 μL. The range of concentrations for different antibiotics, as distributed in 96-well plates, was as follows: 1–1,024 mg/L for CZA (Zavicefta, 2.0 g ceftazidime + 0.5 g avibactam, Italy), 1–16,384 mg/L for ATM (1.0 g, HUNAN KELUN PHARMACEUTICAL Co., Ltd., China), and 1–16,384 mg/L for ceftazidime (TAZIDIME, 0.5 g, China). After incubation for 18 h at 37°C with shaking at 60 rpm, Abs600nm was measured using Multiskan GO. The growth inhibition rate at different concentrations was determined following the formula (Maffioli et al., 2017):

Growth inhibition rate = [Abs600nm of the positive group (without antibiotics)—Abs600nm of the test group (containing antibiotics)]/[Abs600nm of the positive group—Abs600nm of the negative group (without bacteria)].

The antibiotic concentration corresponding to 80% inhibition rate was considered as IC_80_, which was considered as the cut-off to assess antibacterial effectiveness (Supplementary Figure 4D).

### Combined Drug Inhibition Assay

In the 96-well plates, the combination of antibiotics (CZA plus ATM, ATM plus AVI, and CAZ plus AVI) followed the checkerboard dilution method. CAZ and ATM were diluted using the twofold gradient dilution method, and their final concentration reached 0.03125–16,384 mg/L. The concentration of AVI (Avibactam sodium, 5 mg, Shanghai Taosu Biochemical Technology Co., Ltd., China) was kept at 4 mg/L based on the CLSI protocol. After incubation for 18 h at 37°C with shaking at 60 rpm, their Abs600nm values were measured. Further, the combined gradient dilution of AVI and ATM followed the checkerboard dilution method: AVI from 4.0–0.00390 mg/L and ATM from 2.0–0.03125 mg/L.

### Statistical Analysis

Strains containing the same ARGs acted as one subgroup. The intersections of ARGs of strains were analyzed, indicating which ARGs were in which strains or were unique to one strain. Subgroup analysis was performed using the online software Bioinformatics & Evolutionary Genomics^4^. Data analysis and output were performed with GraphPad Prism 6.0. There were at least three biological repeats for the results of each group. The schematic diagram of the materials and methods in our study is shown in Supplementary Figure 1.

## Results

### *Klebsiella pneumoniae* Positive Culture Rate Was the Highest in Patients With IPN in ICUs

**FIGURE 1:**
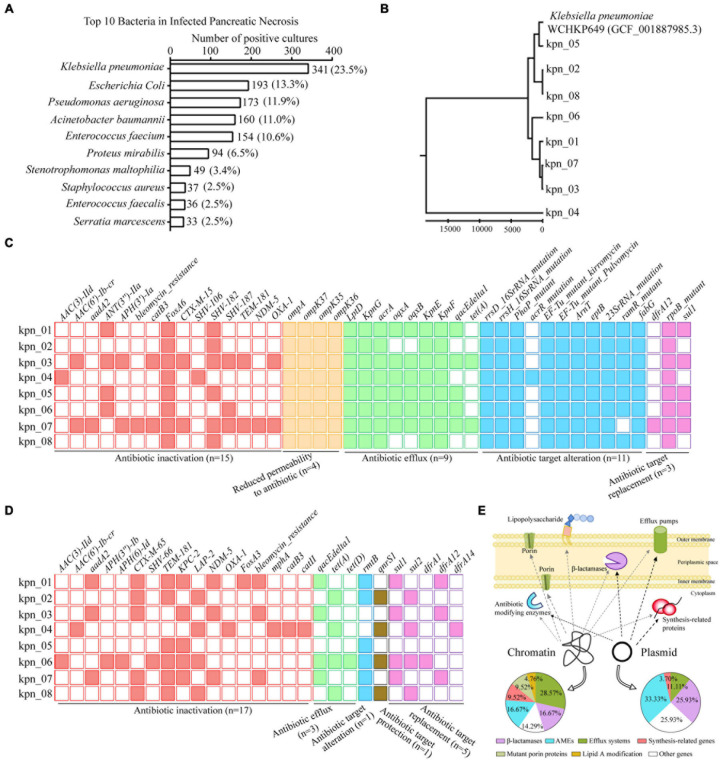
WGS revealed ARGs distribution in genome of *K. pneumoniae* from patients with IPN in the ICU. **(A)** Top 10 bacteria isolated from 758 patients with IPN. **(B)** Evolutionary analysis of *K. pneumoniae* homologous based on all the variant numbers. **(C)** 42 ARGs distribution in chromatin, containing five antibiotics resistance classes. **(D)** 27 ARGs distribution in plasmid. **(E)** Graphic model showed the relationship between ARGs distribution and resistance mechanisms. AMEs, antibiotic modifying enzymes.

We analyzed 1,454 positive cultures of bacterial strains isolated from 758 patients with IPN in our hospital from 2015–2019. It was found that the number of *K. pneumoniae* positive cultures reached 341 (23.5%), ranking first among all bacterial strains. *E. coli* came second with 193 (13.3%), significantly lower than *K. pneumoniae*. In Figure 1A, we showed the top 10 bacteria in IPN, including *Pseudomonas aeruginosa, Acinetobacter baumannii, Enterococcus faecium, Proteus mirabilis, Stenotrophomonas maltophilia, Staphylococcus aureus, Enterococcus faecalis*, and *Serratia marcescens*. Next, we analyzed the polymicrobial infection of these bacteria in 758 patients with IPN. As Supplementary Figure 2A showed, monomicrobial infection of *K. pneumoniae* reached 105 (32.1%), ranking first among all bacterial strains. Among 758 patients with IPN, 327 had one kind of bacteria, 258 had 2 kinds, and 173 had ≥ 3 kinds, suggesting that both monomicrobial and polymicrobial infection occurred in IPN.

### The Distribution and Resistance Characteristics of Antibiotic Resistance Genes Were Represented Through WGS and AST

To explore the antibiotic resistance determinants in the genome of *K. pneumoniae* from IPN, we independently isolated eight *K. pneumoniae* strains from eight patients with IPN to perform WGS. First, to rule out cross-infection during sampling, we performed evolutionary analysis to analyze their homology, indicating that these *K. pneumoniae* genomes were significantly different because of variant SNPs/index (Figure 1B). Next, 42 antibiotic resistance genes (ARGs) were screened out in chromatin (Figure 1C) and 27 ARGs in the plasmid (Figure 1D). In detail, the percentage of β-lactamase was 16.67% in the chromatin and 25.93% in the plasmid, that of antibiotic modifying enzymes (AMEs) was 16.67% in the chromatin and 33.33% in the plasmid, while that of efflux systems was 28.57% in the chromatin and 11.11% in the plasmid; these and other pathway distributions are shown in Figure 1E.

**FIGURE 2:**
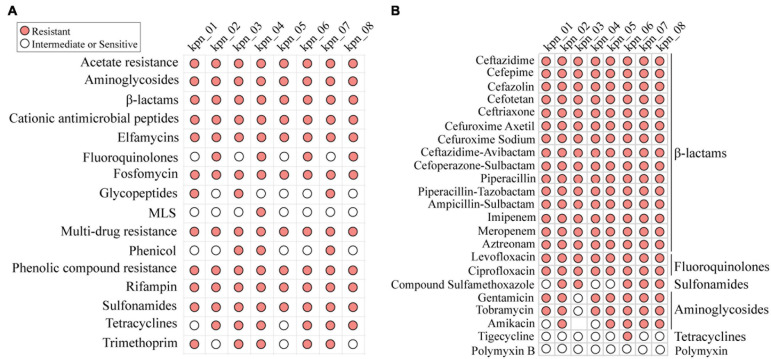
MDR profile of *K. pneumoniae* for antibiotics commonly used in clinic. **(A)** Antibiotic resistance phenotypes based on open database. **(B)** The resistance phenotypes to common antibiotics in clinic, including β-lactams, fluoroquinolones, sulfonamides, aminoglycosides, and tetracyclines. MLS, macrolides-lincosamids-streptogramins.

These isolates were considered MDR through WGS and AST. Based on their genotypes, they should be resistant to known antibiotics in clinics although the phenotype of each isolate was different (Figure 2A). When antibiotics commonly used in clinics, such as β-lactams, fluoroquinolones, aminoglycosides, tetracyclines, and sulfonamides, were chosen to perform AST (Figure 2B), all these isolates were observed to be resistant to β-lactams and fluoroquinolones. Except for kpn_03, the other isolates were resistant to aminoglycosides. In addition, we retrospectively analyzed the antimicrobial susceptibility profile of 758 patients with IPN. Except for CZA, other antibiotics’ AST in *K. pneumoniae* were similar to these eight strains (Supplementary Figure 2B). For antimicrobial susceptibility profiles of other bacteria with a high infection rate, like *K. pneumoniae*, they had high resistance to β-lactams antibiotics (Supplementary Figure 2C).

### CZA Inhibited MDR *Klebsiella pneumoniae* Less Effectively Than CZA Plus ATM

**FIGURE 3:**
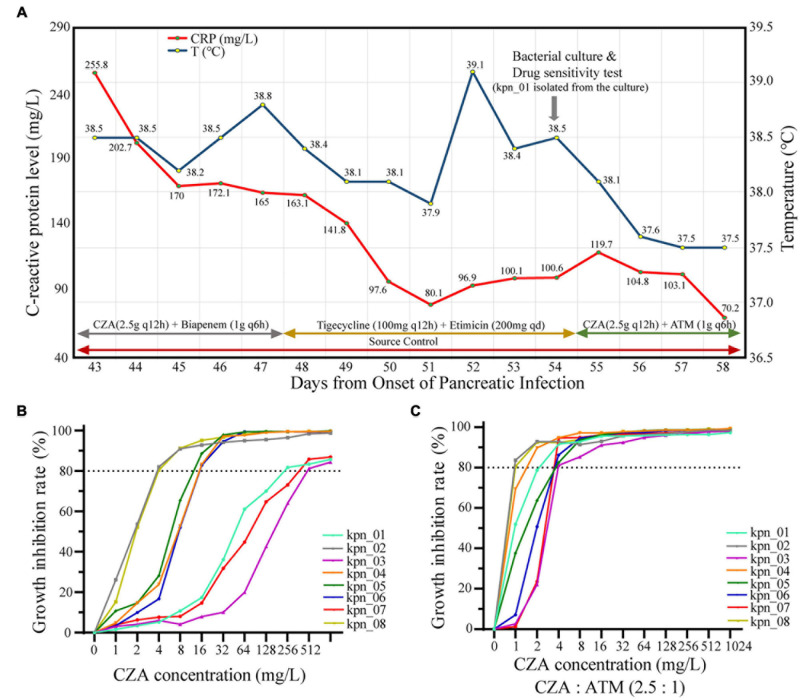
CZA plus ATM had more significant antibacterial effectiveness than CZA *in vivo* and *in vitro*. **(A)** The retrospective analysis for patients with IPN found CZA plus ATM (2.5:1) became the key factor of control of infections. **(B)** The growth inhibition rate of different CZA concentrations showed poor antibacterial effectiveness when 80% inhibition rate was used as the cut-off, and each value expressed the mean of six bio-repeats. **(C)** Inhibition concentration of 80% growth inhibition effectiveness (IC_80_) significantly decreased while CZA plus ATM (2.5:1).

Although CZA as the novel drug was approved in China in 2019, its standalone use did not effectively control most infections in our ICU. As shown in Figure 3A, during the hospitalization of the patient, the body temperature was significantly decreased after adjusting to CZA plus ATM. In contrast, CZA plus biapenem and tigecycline plus etimicin could not control the body temperature as the C-reactive protein level (CRP) decreased. Similarly, *in vitro*, CZA did not effectively inhibit the growth of all *K. pneumoniae*; IC_80_ of 37.5% (3/8) isolates reached ≥ 256 mg/L (Figure 3B). When CZA plus ATM (2.5:1) was administered, their IC_80_ for CZA was ≤ 4 mg/L (Figure 3C), suggesting that CZA plus ATM showed effective antibacterial activity.

### Genomic New Insights and Suitable Treatment Strategies Were Discovered by Combining the Phenotype With the Genotype

**FIGURE 4:**
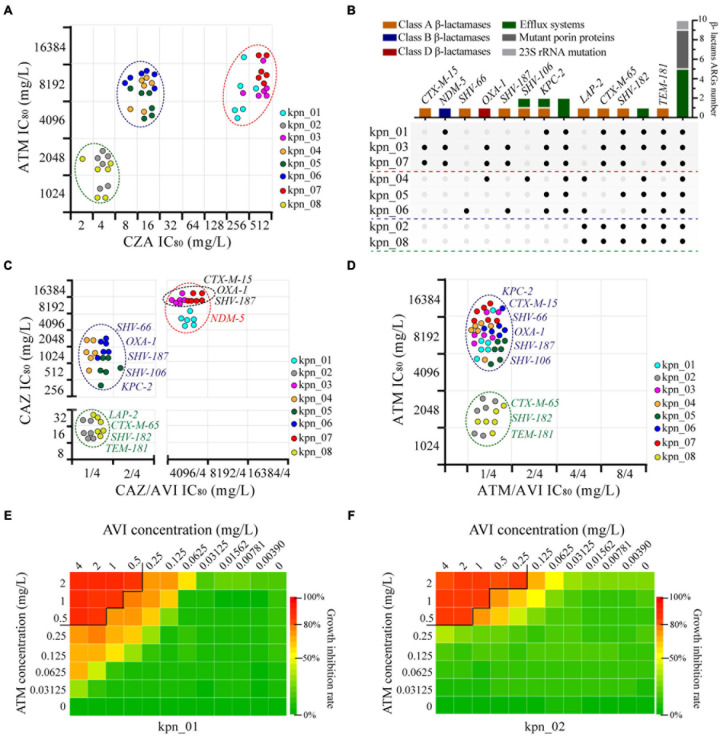
Genomic new insights of MDR *K. pneumoniae* and ATM plus AVI as the best treatment strategy. **(A)** Three different types of phenotypes were observed in these *K. pneumoniae* according to the IC_80_ of CZA and ATM, and each point expressed one bio-repeat. **(B)** 14 subgroups were divided, different colors showed different resistance mechanisms, and the black point indicated the presence of one gene in the isolate. **(C)** Four main types of phenotypes showed the condition of β-lactamases as resistance determinants to CAZ, AVI was maintained at 4 mg/L in CAZ/AVI. **(D)** Two types of phenotypes showed the condition of ESBLs and KPC-2 as resistance determinants to ATM, AVI was maintained at 4 mg/L in ATM/AVI. **(E)** Combined drug inhibition assay for ATM and AVI of kpn_01, IC_80_ as the cut-off. **(F)** Combined drug inhibition assay for ATM and AVI of kpn_02, IC_80_ as the cut-off.

After analyzing for ATM and CZA, we found three different phenotypes, which should result from different genotypes (Figure 4A). We extracted 26 β-lactams-ARGs from all the ARGs from plasmid and chromatin, including one metal-β-lactamase (MBL) (*bla*_NDM–5_), eight extended-spectrum β-lactamases (ESBLs), and two other β-lactamases (*bla*_LAP–2_ and *bla*_KPC–2_) (Figure 4B). By observing the phenotypes for CAZ and CAZ plus AVI (4 mg/L), we found that isolates co-harboring *bla*_NDM–5_, *bla*_CTX–M–15_, *bla*_OXA–1_, and *bla*_SHV–187_ had more than twice as much IC_80_ as isolates with *bla*_NDM–5_ (8,192 vs 4,096 mg/L), and their differences could be wiped out by AVI (4,096 vs 4,096 mg/L). This suggested that *bla*_NDM–5_ was the resistance determinant for IC_80_ of 4,096 mg/L and that *bla*_CTX–M–15_, *bla*_OXA–1_, or *bla*_SHV–187_ further enhanced *bla*_NDM–5_ function (Figure 4C). Moreover, other isolates had two obvious differences for IC_80_ (≥ 256 vs ≤ 32 mg/L), showing that SHV-66, OXA-1, SHV-187, SHV-106, and KPC-2 had stronger hydrolysis of CAZ than LAP-2, CTX-M-65, SHV-182, and TEM-181. AVI also eliminated these differences (CAZ ≤ 2 mg/L); therefore, AVI could inhibit the 10 ARGs. In contrast, for ATM and ATM plus AVI (4 mg/L), the IC_80_ of ATM was only 1 mg/L because of the inhibition function of AVI. For eight ESBLs and KPC-2, which was the resistance determinant to ATM, two distinct clusters appeared following ATM treatment (≥4,096 vs ≤ 2,048 mg/L), indicating that CTX-M-65, SHV-182, and TEM-181 were weaker than KPC-2, CTX-M-15, SHV-66, OXA-1, SHV-187, and SHV-106 in the hydrolysis of ATM (Figure 4D).

Next, we explored the concentration of drugs using combined inhibition assay and found that the minimum effective concentration combination for ATM plus AVI were 0.5 mg/L plus 4 or 2 mg/L plus 0.25 mg/L. Combined drug assay between ATM and AVI was shown in Figures 4E,F and Supplementary Figure 3, providing a reference for the clinical treatment of MDR *K. pneumoniae* as well as anti-infective therapy against complex infections and diseases.

## Discussion

The emergence and spread of MDR bacteria have occurred due to two key factors: irrational antibiotic use and low host immunity (Reverter et al., 2020). These two factors have been found to be particularly prominent in ICUs, especially during the on-going COVID-19 pandemic (Getahun et al., 2020). To ensure the scientific and rational use of antibiotics, genomic studies are being increasingly conducted (Baker et al., 2018). *Bla*_NDM–5_ was first identified by sequencing (Hornsey et al., 2011), and the gene *gar* was identified as a novel ARG to aminoglycoside plazomicin through metagenomics (Bohm et al., 2020). Moreover, WGS clarified that *bla*_OXA–232_ enhances the resistance and virulence of *bla*_NDM–1_ (Lee et al., 2020). WGS also indicated the emergence of *K. pneumoniae* strain ST11, co-harboring *bla*_NDM–5_, *bla*_CTX–M–15_, and *bla*_OXA–1_, as a new superbug with high resistance and virulence in Beijing, China (Zhao et al., 2020). In our study, WGS was used to analyze ARG distribution in MDR *K. pneumoniae* isolated from IPN samples collected from patients admitted to the ICU of our pancreatitis center (China’s largest pancreatitis treatment referral center). Using MLST analysis and based on the phenotypes and genotypes, the isolates kpn_03 and kpn_07, with the highest resistance to CZA, were considered *K. pneumoniae* strain ST11 co-harboring *bla*_NDM–5_, *bla*_CTX–M–15_, and *bla*_OXA–1_, thus supporting the assumption that the emerging *K. pneumoniae* strain may have spread in China. In addition, we showed that the phenotypes of the ESBLs enhanced the resistance of MBLs, suggesting that the co-existence of ESBLs and MBLs has become a new challenge for anti-infective drugs globally, and we first clarified that *bla*_CTX–M–65_, *bla*_SHV–182_, and *bla*_TEM–181_ had relatively lower resistance to β-lactams than other ESBLs. However, our results provide novel evidence for the emergence and spread of MDR *K. pneumoniae*, highlighting the need for the scientific and rational use of antibiotics in patients with complex critical diseases admitted to ICUs.

Bacterial infections in ICUs are the leading cause of mortality in hospitalized patients, and nearly half of ICU patients worldwide experience such infections (O’Grady and Kadri, 2017). For some complex severe diseases, infections are often difficult to control due to resistant bacteria, and this situation has amplified during the COVID-19 pandemic. MBL-producing *K. pneumoniae* and methicillin-resistant *Staphylococcus aureus* increased the difficulty in the treatment of COVID-19 (Sharifipour et al., 2020; Arteaga-Livias et al., 2021). Similar to COVID-19, in IPN, *K. pneumoniae* was the main pathogen, which had the second highest positive culture rate, after *E. coli*, for gram-negative bacteria (Garret et al., 2020). Our study indicated that *K. pneumoniae* (341/1454, 23.5%) had the highest positive culture rate in patients with IPN in China, and there was also a high rate of MDR. Except for antibiotic overuse and misuse, environments related to nosocomial infections may be another factor for this phenomenon. This is because our pancreatitis center in the ICU was a separate ward that only received patients with pancreatitis where the probability of cross-infection was relatively higher for MDR bacteria than other wards with high mobility. However, this study not only provided a new understanding of IPN but also provided a reference for treating other bacterial infections in ICUs.

However, CZA administration may no longer be applicable to the current situation with the prevalence of MBL-producing bacteria, and CZA overuse is one of the main reasons for the prevalence of MBL-producing bacteria (Papadimitriou-Olivgeris et al., 2019). The administration of CZA plus ATM *in vivo* has been proven to have suitable anti-infective effects on MBL-producing bacteria (Falcone et al., 2020), and the administration of ATM plus AVI *in vitro* has also been reported to inhibit the growth of MBL-producing bacteria (Singh et al., 2015). In the present study, we confirmed these reports *in vivo* and *in vitro*. We showed that ATM plus AVI also had an adequate inhibitory effect on *K. pneumoniae* strain ST11 co-harboring *bla*_NDM–5_, *bla*_CTX–M–15_, and *bla*_OXA–1_, which has been spreading in China, and reported the lowest effective dose of ATM and AVI. These results will further promote the rational use of antibiotics in ICUs.

For antibiotic susceptibility testing, the Mueller-Hinton broth (MHB) has been recommended by CLSI as an appropriate medium because of its convenience and stability; therefore, we used it to perform AST in clinics (Figure 2B). However, in the checkerboard dilution method used to analyze the drug synergy and growth inhibition curve, we chose the Luria-Bertani (LB) broth as the medium instead of MHB, which was based on three key reasons. First, LB broth has long been considered the best medium for the growth of gram-negative bacteria (Sezonov et al., 2007), and we showed that the 24 h growth and standard curves of *K. pneumoniae* were similar to those of *E. coli* (Supplementary Figures 4A,B). Second, because the stability of some key ARGs located on plasmids was the main factor to maintain antibiotic resistance (Wein et al., 2019), we chose LB broth as the medium where plasmid DNA could be copied suitably. Third, it has been reported that the same compound exhibits different results under drug synergy testing between LB broth and MHB (Pormohammad and Turner, 2020). The LB medium used in our study was used to confirm the stable effectiveness of ATM plus AVI, irrespective of the medium (whether LB or MHB). This is because ATM plus AVI has been reported to show antibacterial effects in MHB (Chauzy et al., 2019). In contrast, although the half-life of β-lactams in LB broth was lower than that in MHB (Brouwers et al., 2020), we found that LB broth did not determine the resistance against CAZ, ATM, and CZA in our study because the absorbance of 1 or 2 mg/L antibiotics with *E. coli* at 600 nm was the same as that of the blank control (LB broth) after 18 h of incubation at 37°C (Supplementary Figure 3C). In terms of methods, our study provided a new understanding of antibiotic resistance.

In conclusion, our study revealed that novel ARG distribution was related to common clinical antibiotic resistance in ICU patients with IPN. After combining phenotypes, we showed some new genomic insights and supported the proposal that ATM plus AVI should be considered the main treatment for MDR *K. pneumoniae* in ICUs. These results will not only reduce the risks associated with antibiotic misuse and overuse in ICUs but will also improve the prognosis of complex infectious diseases.

## Data Availability Statement

The genomes of kpn_01, kpn_02, kpn_03, kpn_04, kpn_05, kpn_06, kpn_07 and kpn_08 have been deposited in Genbank under BioSample no. SAMN18042951, SAMN18042952, SAMN18042953, SAMN18042954, SAMN18042955, SAMN1804 2956, SAMN18042957, and SAMN18042958, respectively.

## Ethics Statement

Written informed consent was obtained from the individual(s) for the publication of any potentially identifiable images or data included in this article.

## Author Contributions

WL and ZT conceived and designed the study. HH, YLu, and PW performed the experiments *in vitro*. WW and JC performed the antimicrobial susceptibility testing. YLi and KG collected clinical data. LH carried out genome sequencing. HH, KG, and LH performed data analysis. YLi and SL participated in discussion and data analysis. WL, HH, KG, and YLu wrote the manuscript. All authors read and approved the final manuscript.

## Conflict of Interest

LH was employed by the company “Department of Bioinformatics, Hugobiotech Co., Ltd., Beijing, China.” The remaining authors declare that the research was conducted in the absence of any commercial or financial relationships that could be construed as a potential conflict of interest.

## Footnotes

1 https://github.com/lh3/bwa/archive/v0.7.17.tar.gz

2 https://github.com/rrwick/Filtlong

3 https://github.com/tseemann/abricate

4 http://bioinformatics.psb.ugent.be/webtools/Venn/

## Supplementary Material

Supplementary Figure 1 Schematic diagram of the materials and methods in our study.

Supplementary Figure 2 Microbial infection and antimicrobial susceptibility profile of 758 patients with IPN. **(A)** Infection distribution map of 758 patients, **(B)** Retrospective analysis of *K. pneumoniae* AST, **(C)** Retrospective analysis of AST for *Escherichia Coli, Pseudomonas aeruginosa*, and *Acinetobacter baumannii.*

Supplementary Figure 3 Combined drug inhibition assay for ATM plus AVI. Panel **(A)** for kpn_03, panel **(B)** for kpn_04, panel **(C)** for kpn_05, panel **(D)** for kpn_06, panel **(E)** for kpn_07, panel **(F)** for kpn_08.

Supplementary Figure 4 The growth characteristics of *K. pneumoniae* in Lysogeny Broth (LB) medium were similar to *E. coli in vitro*. **(A)** Bacteria growth curve over 24 h *in vitro* for *K. pneumoniae* and *E. coli*. **(B)** Standard curve of concentration and absorbance at 600 nm. **(C)** Test of the validity of CAZ, ATM, and CZA. **(D)** From visual inspection, an 80% inhibition rate for *K. pneumoniae* could be considered as the suitable inhibition effectiveness, *E. coli* as the control.
