# Supplementary figures and images for: Genomic New Insights Into Emergence and Clinical Therapy of Multidrug-Resistant Klebsiella pneumoniae in Infected Pancreatic Necrosis
Source: Front Microbiol. 2021 Jun 25;12:669230. doi: 10.3389/fmicb.2021.669230 (PMC8269854; doi:10.3389/fmicb.2021.669230)

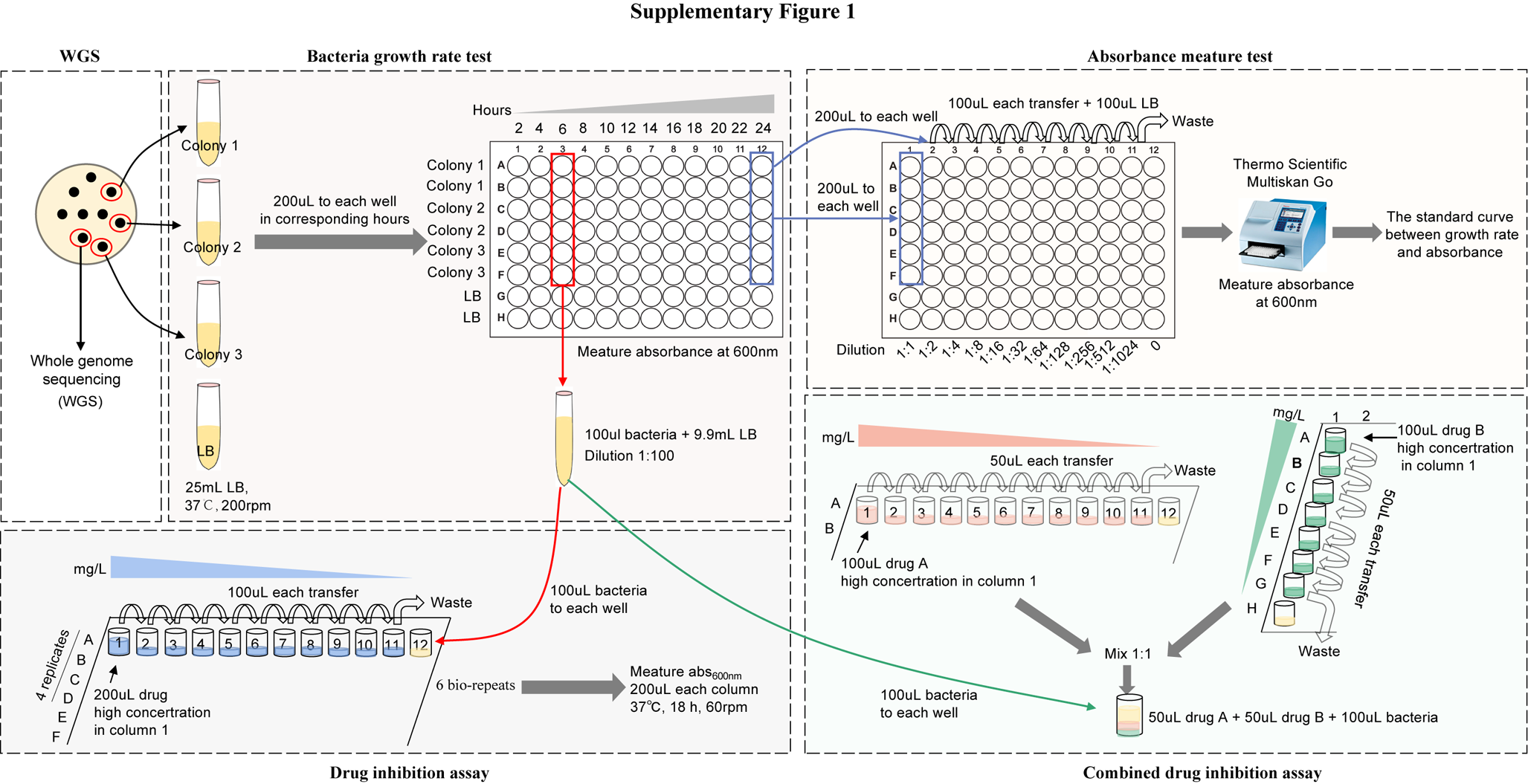

Supplement: Supplementary Figure 1 — Schematic diagram of the materials and methods in our study. [file Image_1.TIF]

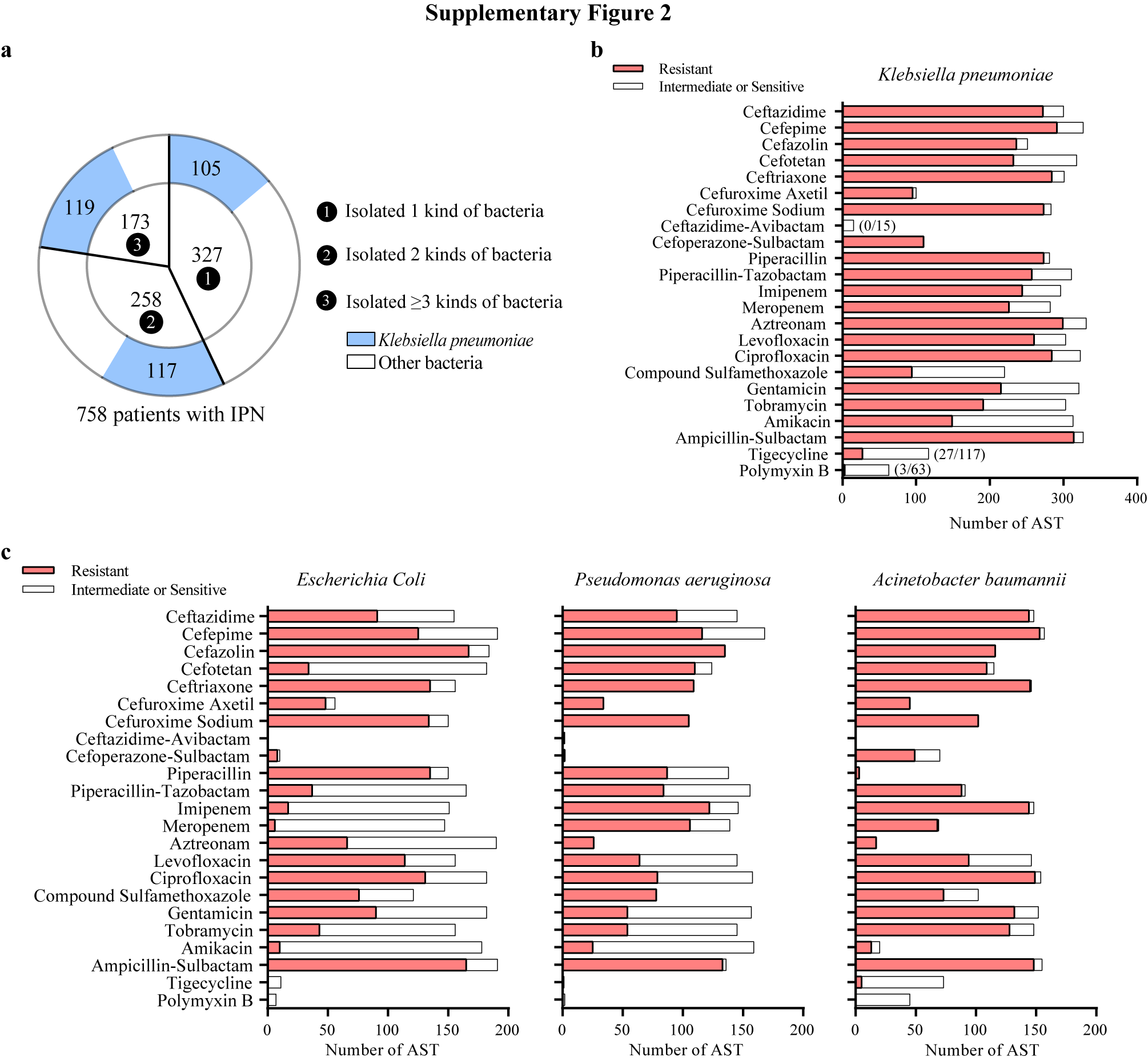

Supplement: Supplementary Figure 2 — Microbial infection and antimicrobial susceptibility profile of 758 patients with IPN. (A) Infection distribution map of 758 patients, (B) Retrospective analysis of K. pneumoniae AST, (C) Retrospective analysis of AST for Escherichia Coli, Pseudomonas aeruginosa, and Acinetobacter baumannii. [file Image_2.TIF]

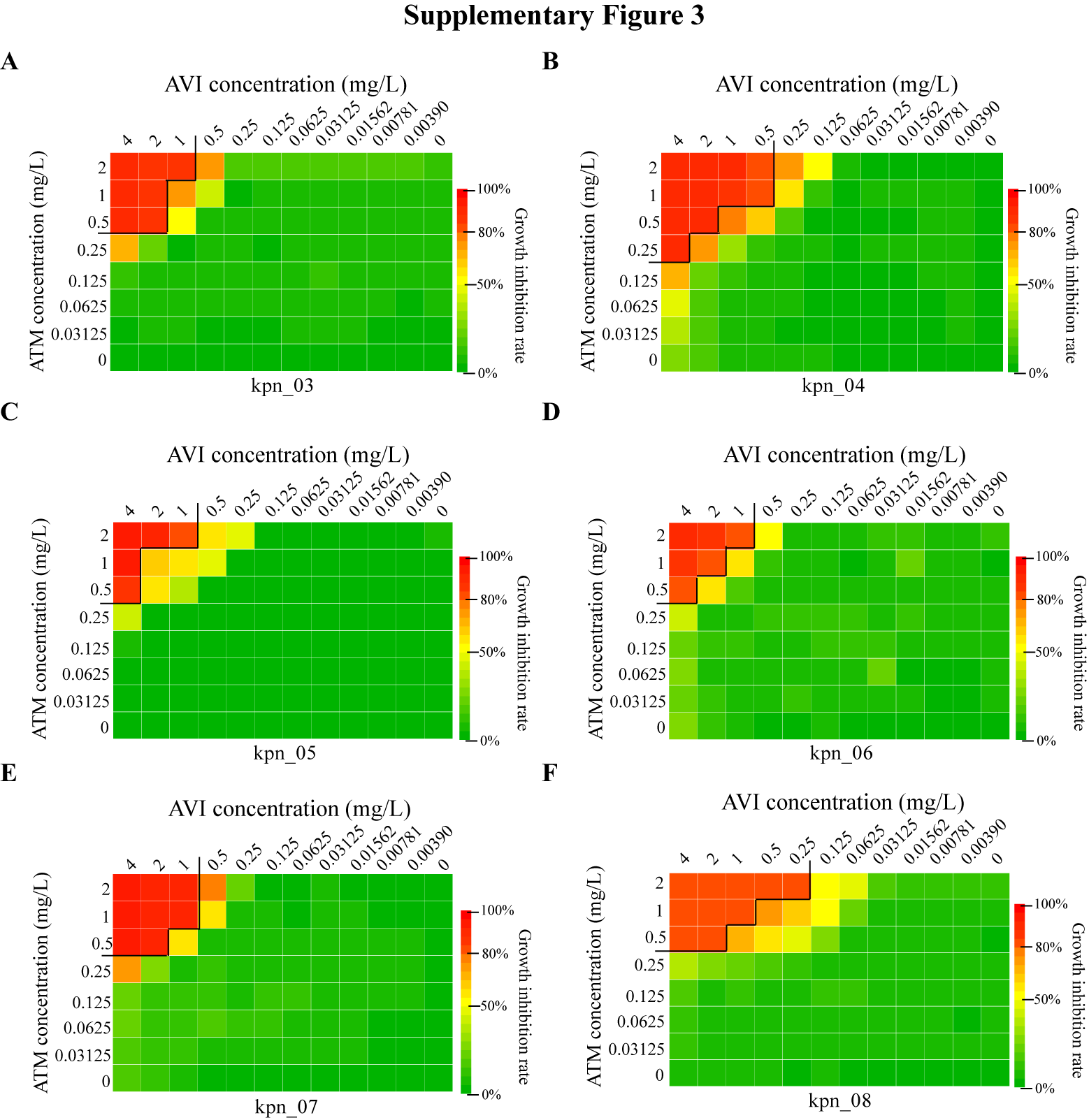

Supplement: Supplementary Figure 3 — Combined drug inhibition assay for ATM plus AVI. Panel (A) for kpn_03, panel (B) for kpn_04, panel (C) for kpn_05, panel (D) for kpn_06, panel (E) for kpn_07, panel (F) for kpn_08. [file Image_3.TIF]

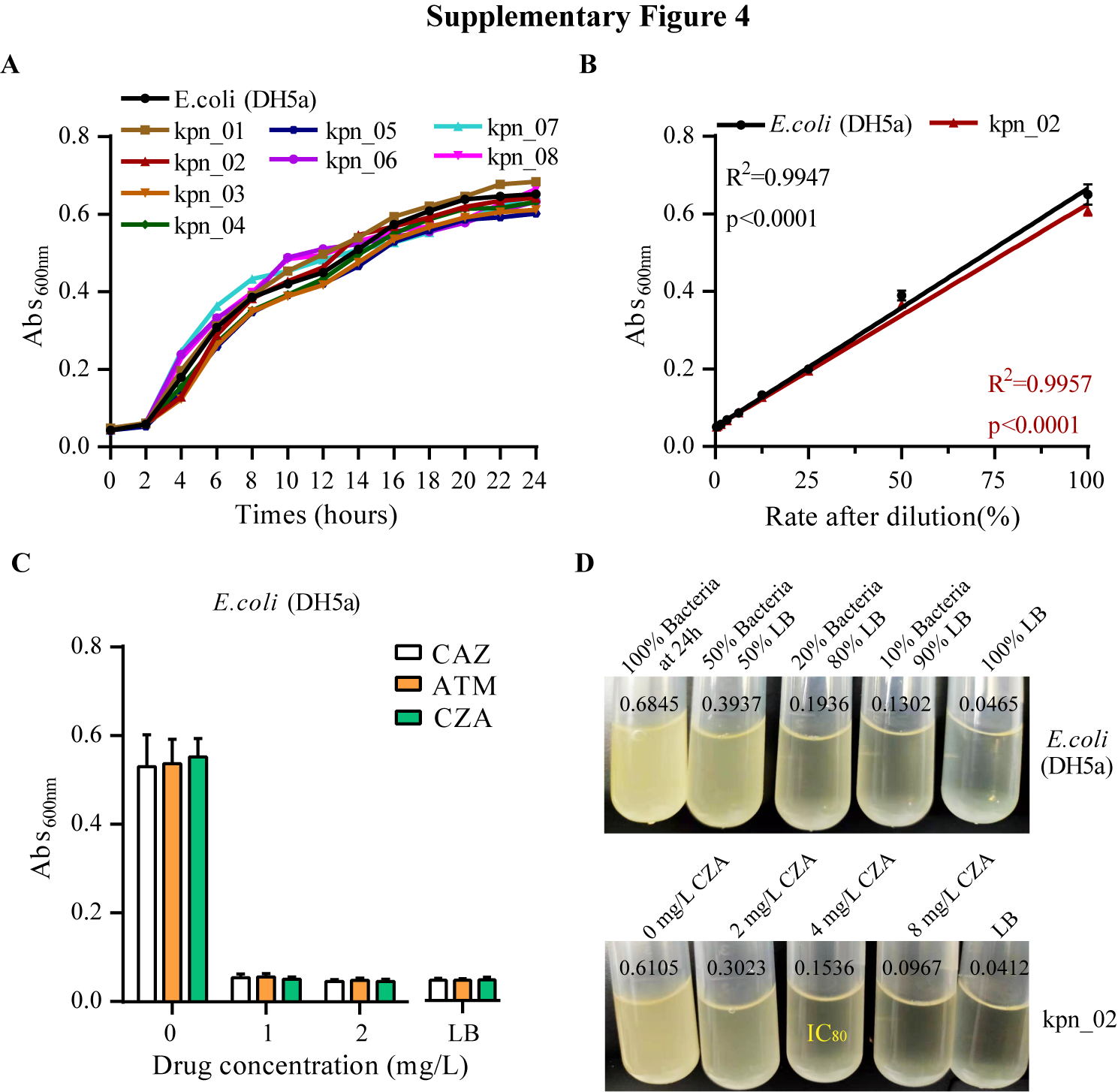

Supplement: Supplementary Figure 4 — The growth characteristics of K. pneumoniae in Lysogeny Broth (LB) medium were similar to E. coli in vitro. (A) Bacteria growth curve over 24 h in vitro for K. pneumoniae and E. coli. (B) Standard curve of concentration and absorbance at 600 nm. (C) Test of the validity of CAZ, ATM, and CZA. (D) From visual inspection, an 80% inhibition rate for K. pneumoniae could be considered as the suitable inhibition effectiveness, E. coli as the control. [file Image_4.TIF]
